# Alcohol Consumption and Cancer Risk: Two Sample Mendelian Randomization

**DOI:** 10.3390/epidemiologia5030043

**Published:** 2024-09-17

**Authors:** Yongho Jee, Mikyung Ryu, Jae-Woong Sull

**Affiliations:** 1 Advanced Biomedical Research Institute, Ewha Womans University Seoul Hospital, Seoul 07804, Republic of Korea; jyongho@ewha.ac.kr; 2 Institute of Genetic Epidemiology, Basgenbio Inc., Seoul 04167, Republic of Korea; 3 Department of Biomedical Laboratory Science, College of Health Science, Eulji University, Seongnam 13135, Republic of Korea; jsull@eulji.ac.kr

**Keywords:** alcohol consumption, causality, Mendelian randomization, cancer

## Abstract

Although numerous observational studies have reported on the association between alcohol consumption and cancer, insufficient studies have estimated the causality. Our study evaluated the causal relationship between various types of cancer according to the frequency of drinking and the amount of alcohol consumed. The research data were obtained from the publicly available MR-Base platform. The frequency and amount of drinking were selected as the exposure, and 16 cancer types were selected as the outcome. Two-sample summary data Mendelian randomization (2SMR) was conducted to examine the causality between alcohol consumption and cancer type. Additionally, for cancers suspected of pleiotropy, outliers were removed and re-analyzed through radial MR. The MR results using the inverse variance weighted (IVW) method were different before and after removing outliers. The biggest differences were found for esophageal cancer and biliary tract cancer. For esophageal cancer, after removing outliers (rs13102973, rs540606, rs650558), the OR (95% CI) was 3.44 (1.19–9.89), which was statistically significant (*p* = 0.02172). Even in biliary tract cancer, after removing outliers (rs13231886, rs58905411), the OR (95% CI) was 3.86 (0.89–16.859), which was of borderline statistical significance (*p* = 0.07223). The strongest association was found for esophageal cancer. For other cancers, the evidence was not sufficient to draw conclusions. More research is needed to understand the causality between drinking and cancer.

## 1. Introduction

Heavy alcoholic beverage consumption is an established risk factor for poor health outcomes, and it is one of the leading causes of death [1]. Heavy alcohol consumption is a well-documented deleterious lifestyle behavior that activates the brain’s reward pathways, resulting in the release of dopamine [2]. Alcohol consumption is linked to cancer through several mechanisms: acetaldehyde, a byproduct of ethanol metabolism, causes DNA damage; oxidative stress from reactive oxygen species (ROS) leads to cellular damage; and immunosuppression and inflammation promote tumor growth. Additionally, alcohol increases estrogen levels, raising the risk of hormone-sensitive cancers, and induces folate deficiency, resulting in DNA instability. These pathways are supported by extensive literature, including reviews [3,4,5,6,7].

Evidence that drinking alcohol is a risk factor for cancer has been reported since the early 20th century [8]. According to the IARC Monographs reported in 1988, alcoholic beverages were described as “carcinogenic to humans”. A few decades later, an association between alcohol consumption with cancers of the oral cavity, pharynx, larynx, esophagus, liver, colorectum, and female breast were reported [9]. Alcohol consumption is known to elevate the risk of cancer by increasing the level of oxidized metabolite acetaldehyde, which is carcinogenic to humans [10]. However, alcohol consumption may simultaneously reduce the cancer risk by alternative mechanisms, such as by increasing insulin sensitivity through elevated adiponectin levels [11].

In observational studies, alcohol consumption was reported to be positively correlated with the risk of head and neck cancer, esophageal cancer, stomach cancer, liver cancer, and breast cancer [12,13,14,15], while non-Hodgkin’s lymphoma [14,15,16] is inversely related. Additionally, heavy drinking was associated with an increased risk of colorectal cancer, whereas light or moderate drinking was associated with a reduced risk [17]. Results from previous observational studies can be interpreted as identifying associations, but their causality interpretation is limited due to the influence of confounding variables. However, recently, through the Mendelian randomization research method, which looks at the relationship with cancer occurrence through the amount of alcohol exposure as if it were a genetic variant, it has become possible to interpret it from a causal point of view. Genetic factors are randomly assigned and determined at conception, so they are independent of confounding variables. The new research method developed using this perspective is called Mendelian randomization.

In this study, a two-sample Mendelian randomization study was conducted to investigate the causal relationship between drinking frequency/alcohol consumption and the occurrence of various cancers.

## 2. Materials and Methods

### 2.1. Data Source

The data of this study were obtained from the MR-Base platform (http://www.mrbase.org, accessed on 12 March 2023), which systematically integrates the GWAS summary datasets. For the exposure, we used data on drinking frequency [18] and drinking amount [19]. Alcohol consumption data were measured in units of weekly alcohol consumption. We selected GWAS datasets that specifically investigated alcohol consumption as the exposure and various types of cancer as the outcomes. This ensured that the genetic instruments (single nucleotide polymorphisms, or SNPs) used in the MR analysis were relevant and directly related to our research question. Aiming to minimize heterogeneity and population stratification bias, we selected GWAS datasets conducted in similar populations. For this study, we used data from predominantly Western populations.

### 2.2. Variables

The data for alcohol consumption were obtained from the GWAS conducted by Howe et al. [20] under the Within Family GWAS Consortium, which provided a comprehensive analysis of genetic associations with alcohol consumption. The dataset used for this study is available on the MR-Base platform (http://www.mrbase.org) under the dataset ID ieu-b-4834 [20]. This citation acknowledges both the original study and the data repository from which the data were accessed, ensuring proper credit is given to the researchers and the platform facilitating the data availability (MRcIEU). We used “Alcohol consumption” (ID: ieu-b-4834) and “Alcohol frequency” (ID: ukb-a-25) as search terms for our analysis. As an outcome, the cancer types were stomach, liver, lung, thyroid, cervix, breast, prostate, colorectal, bladder, larynx, lip/oral/pharynx, brain, biliary tract, and esophagus. For both exposure and outcome data, sample size, number of variants, first author, year, name of consortium, sex, and build reported in the MR-Base were extracted.

### 2.3. Statistical Analysis: Two-Sample Mendelian Randomization Analysis

We confirm that the exposure (alcohol consumption) and outcome (cancer risk) datasets used in our study were obtained from separate and independent sources. To further ensure the independence of the datasets, we cross-referenced participant identifiers and confirmed that there was no overlap in the individual participants included in both the exposure and outcome datasets.

After selecting each exposure and outcome using the MR-Base platform, each two-sample Mendelian randomization was analyzed. SNPs obtained from the GWAS of exposure and outcome were used with clumping to prune the SNPs for LD. If a particular exposure SNP was not present in an outcome dataset, proxy SNPs were used instead through LD tagging.

SNPs were selected based on a significance threshold of *p* < 5 × 10^−8^. This stringent threshold ensures that only SNPs with a strong association with the exposure were included, reducing the risk of including weak or non-informative instruments. To ensure the reliability of the selected SNPs as proxies for alcohol consumption and frequency, we applied several validation steps. First, we conducted a clumping procedure to remove the SNPs in linkage disequilibrium (LD) to avoid redundancy. Second, we cross-referenced the selected SNPs with the literature and database annotations to confirm their known associations with alcohol-related traits. Finally, we assessed the validity of these SNPs in our MR analysis by evaluating their relevance and strength as instruments through standard MR diagnostics.

### 2.4. Outlier Detection

Outliers were identified using the radial MR framework, which provides a measure of heterogeneity through the radial distance statistic. Specifically, we used the radial Q-statistic to quantify the influence of each genetic variant on the overall Mendelian randomization analysis. A genetic variant was classified as an outlier if its radial Q-statistic exceeded a threshold of 3 standard deviations from the mean radial Q-statistic of all variants. This threshold was chosen to balance sensitivity and specificity in detecting influential outliers without overfitting the model. Removing outliers is crucial to reduce the impact of variants that disproportionately affect the MR estimates due to pleiotropy or other biases. The radial MR method allows us to pinpoint these influential variants more precisely, ensuring the robustness and reliability of our results. By setting a threshold based on the standard deviation of the radial Q-statistic, we aimed to systematically and objectively identify and remove variants that could skew the analysis.

In this case, the default value 0.8 was used as the Minimum LD R^2^ value. For the MAF threshold for aligning palindromes, a default value of 0.3 was used. In this analysis, four methods were selected for two-sample Mendelian randomization: inverse variance weighted, MR-Egger, weighted median, and weighted mode. If the *p*-value of MR-Egger was significant, the pleiotropy test was performed through the intercept test. In addition, when an outlier was suspected, additional analysis was performed through radial Mendelian randomization. In this case, summary statistics were downloaded from the MR-Base platform and additional analysis was performed through R analysis. That is, the results of beta values were converted to odds ratios and 95% confidence intervals and presented as independent figures.

## 3. Results

Table 1 shows the characteristics of the exposure data and outcome data used in this study. In this study, the exposure data were alcohol consumption. Outcome data were extracted from all 16 cancers. All data were from the European Population with HG19/GRCh37 Build.

Supplemental Materials Tables S1 and S2 show the two-sample MR results before removing outliers between alcohol consumption, alcohol intake frequency as an exposure and the 16 cancer types. The MR results by the inverse variance weighted method were not significant in all cancers except stomach cancer, which showed borderline significant results (*p* = 0.0486).

In Table 2 and Table 3 we made a comparison between the two-sample MR results before removing outlier SNPs and after removing outlier SNPs for both alcohol consumption and alcohol intake frequency as an exposure. The MR results by the IVW method were different before and after removing outliers. The largest differences were found for esophageal cancer and biliary tract cancer. For esophageal cancer, after removing outliers (rs13102973, rs540606, rs650558), the OR (95% CI) was 3.44 (1.19–9.89), which was statistically significant (*p* = 0.0217). Even in biliary tract cancer, after removing outliers (rs13231886, rs58905411), the OR (95% CI) was 3.86 (0.89–16.859), which was of borderline statistical significance (*p* = 0.0722).

**Figure 1:**
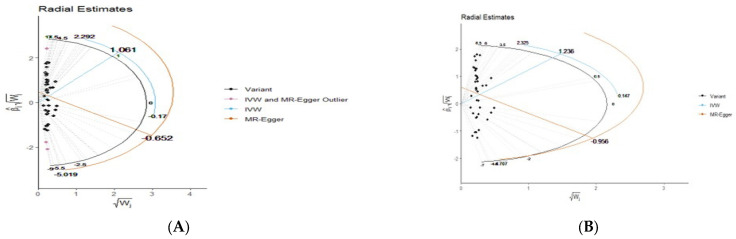
(**A**) Radial MR results for esophageal cancer and outlier discovery (pink colored dots). (**B**) Radial MR results for esophageal cancer after removing outliers.

**Figure 2:**
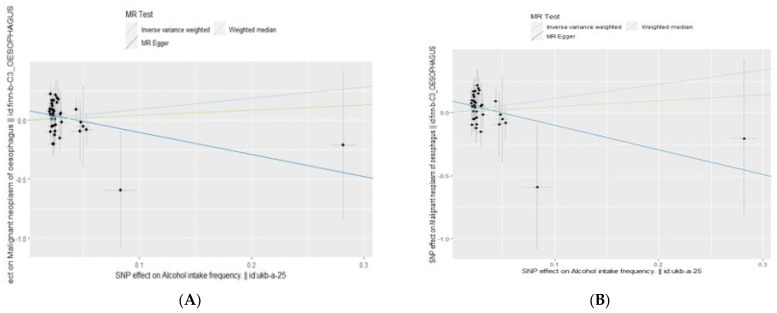
(**A**) Scatter plot for esophageal cancer before removing outliers. (**B**) Radial MR results for esophageal cancer after removing outliers.

Figure 1A shows the existence of outliers through radial MR. The orange color in Figure 1A indicates outliers. Figure 1B is the radial MR result after removing the outliers. Figure 2A,B compares the scatter plot before and after removing three outliers from esophageal cancer.

## 4. Discussion

In this study, two-sample MR analysis was conducted using the MR-Base platform aiming to estimate the causal relationship between alcohol consumption and cancer risk, which is still controversial throughout previously reported observational studies [6,7]. One alcohol-related exposure and 16 cancer types were analyzed as outcomes. This study used Western data for both exposure and outcome.

Results of the study IVW method results were different before and after removing outliers. The biggest differences were found in esophageal cancer and biliary tract cancer. For esophageal cancer, after removing outliers (rs13102973, rs540606, rs650558), the OR (95% CI) was 3.44 (1.19–9.89), which was statistically significant (*p* = 0.02172). As an in-depth analysis, since there were 41 SNPs in esophageal cancer, three outliers were found as a result of additional radial MR. That is, this is an analysis of the number of esophageal cancers in 232 people. If the sample of esophageal cancer becomes larger, it is highly likely to be a significant result.

A review of published Western studies on alcohol consumption and cancer is as follows. Genetically predicted alcohol intake was statistically significantly associated with lung cancer in the International Lung Cancer Consortium (OR 1.94; 95% CI 1.41–2.68; *p* = 4.68 × 10^−5^), but UK Biobank (OR 1.12, 95% CI 0.65–1.93, *p* = 0.686) did not show a similar relationship [18]. Alcohol consumption has also been reported to be associated with breast cancer risk in a dose-response manner, with an 8% to 12% increase in risk for every 10 g/day increase in alcohol consumption [25,26]. The detrimental effects of heavy drinking on colorectal cancer were suggested by studies using the COLCA1/COLCA2 and ALDH2 genotypes as indicators of alcohol exposure [17].

Conversely, light alcohol consumption was not associated with an increased risk of colorectal cancer [26] in some studies. Another MR study found no association between alcohol intake and the incidence of prostate cancer [27]. However, there was a statistically significant relationship between alcohol intake and head and neck cancer [28]. However, in this study, there was no statistically significant association between alcohol consumption and other sites of cancer. That is, we found no evidence to support a relationship between alcohol consumption and overall or site-specific cancer risk.

The association between alcohol consumption and cancer risk has been examined extensively in observational studies, revealing varying patterns across different types of cancer. Notably, alcohol consumption has consistently shown positive correlations with the risk of head and neck cancer, esophageal cancer, stomach cancer, liver cancer, and breast cancer [14]. Conversely, other studies have suggested an inverse relationship between alcohol consumption and the risk of kidney cancer [29] and non-Hodgkin’s lymphoma [30,31]. Furthermore, the relationship between alcohol consumption and colorectal cancer risk appears to vary by drinking pattern. Heavy alcohol consumption has been associated with an increased risk of colorectal cancer, whereas light or moderate drinking has been linked to a reduced risk [17]. Larsson et al. also conducted a Mendelian randomization (MR) analysis examining the associations between smoking, alcohol consumption, and cancer risk. They found that genetically predicted alcohol consumption was statistically significantly associated with an increased risk of lung cancer but was not associated with other site-specific cancers or overall cancer risk [18]. In contrast, our study employed a radial MR analysis to compare the results before and after removing outlier SNPs, thereby providing more robust causal estimates [30].

Given that much of the current evidence comes from observational epidemiological studies, an assessment of the causal relationship of these findings is necessary.

The possible mechanisms of alcohol consumption on cancer development are well known. Among the ADH and ALDH enzymes that affect the metabolism of alcohol that enter the body after drinking, the gene that affects ALDH is ALDH2, and it is located on chromosome 12. This gene is an Asian specific gene and is mainly distributed in East Asian countries. In Asians, in the case of a specific type (AA) of ALDH2, facial flushing occurs after drinking, and nausea and vomiting occur. Studies have shown that these subjects are at higher risk of developing esophageal cancer if they continue to drink. On the other hand, in the case of Westerners, it is known that the alcohol-related gene is located on chromosome 4. The main cause of cancer is acetaldehyde, one of the toxins produced by the breakdown of alcohol. Once ingested, alcohol is metabolized by enzymes including alcohol dehydrogenase (ADH), cytochrome P-450 2E1 (CYP2E1) and bacterial catalase to produce acetaldehyde [31]. Acetaldehyde rapidly binds to DNA and proteins and produces DNA adducts, leading to DNA mutations, DNA crosslinking and chromosomal abnormalities [32,33]. Because acetaldehyde is highly reactive with DNA, it can bind to DNA and form DNA adducts that alter its physical conformation and potentially block DNA synthesis and repair [27,28,30]. These DNA adducts are particularly genotoxic because they can cause DNA point mutations, double-strand breaks, sister chromatid exchanges, and conformational changes to chromosomes [32,33].

The limitation of this study is that it is difficult to apply the research results to East Asians. Genetic variations such as the ALDH2 polymorphism, which is more prevalent in East Asian populations, may show different effects on the cancer risk caused by alcohol consumption. Regarding heterogeneity among cancers, cancers have variable etiologies even within the same organ site. Thus, our study was not able to fully account for the heterogeneity with cancer types, potentially obscuring subtype-specific associations. Also, we acknowledge the possibility of horizontal pleiotropy, where genetic variants used as instruments might affect cancer risk through pathways other than alcohol consumption.

We used the methods used to detect and mitigate pleiotropic effects, such as MR-Egger regression, and the use of multiple instruments.

Since our study used diverse data sources, study designs and population characteristics between the studies used for MR analysis can introduce inconsistencies and affect the comparability of results. Due to these limitations, it is crucial to interpret the findings with caution and we emphasize the need for further research, including studies with larger sample sizes and more diverse populations.

## 5. Conclusions

In conclusion, the strongest association was shown for esophageal cancer. For the other cancers, the evidence was not sufficient to draw any conclusions. More research is needed to understand the causality between drinking and cancer. The absence of a significant causal relationship with drinking alcohol in most cancers except for esophageal cancer does not mean that drinking is not harmful. Clearly, in observational studies, it is true that drinking a lot of alcohol was associated with a higher risk of developing various cancers. It is true that alcohol itself is not causal to the occurrence of cancer, but the act of drinking itself increases the risk of cancer, since drinking tends to be accompanied by specific situations. Ultimately, from a public health point of view, it is important to avoid excessive drinking. Future studies should focus on elucidating the underlying biological mechanisms, particularly for esophageal and biliary tract cancers. Additionally, examining the role of genetic predisposition in modulating these risks can provide deeper insights into personalized prevention strategies. Overall, our study underscores the importance of integrating genetic data with epidemiological research to inform public health recommendations and clinical interventions aimed at reducing alcohol-related cancer burdens.

## Figures and Tables

**Table 1:** General characteristics of study data.

	Trait Name	Sample Size	Number of Variants	First Author	Year	Consortium	Sex
Exposure							
ukb-a-25	Alcohol intake frequency	336,965	10,894,599	Neale BM [21]	2017	Neale lab	M, F
ieu-b-4834	Alcohol consumption	83,626	7,914,362	Howe LJ [20]	2022	WFGC	M, F
Outcome	Cancer site						
finn-b-c3-stomach	Stomach	NA	16,380,305	NA	2021	Finngen [22]	M, F
finn-b-C3-liver	Liver	NA	16,380,303	NA	2021	Finngen [22]	M, F
ieu-b-4955	Lung	374,687	11,078,115	Burrows [23]	2021	UK Biobank	M, F
finn-b-c3-colon	Colon	NA	16,380,317	NA	2021	Finngen [22]	M, F
finn-b-c3-thyroid	Thyroid	NA	16,380,316	NA	2021	Finngen [22]	M, F
finn-b-c3-cervix	Cervix	NA	16,378,927	NA	2021	Finngen [22]	M, F
ieu-a-1168	Breast	33,832	13,011,123	Michailidou K [24]	2015	BCAC	F
finn-b-c3-prostate	Prostate	NA	16,377,987	NA	2021	Finngen [22]	M
finn-b-c3-colorectal	Colorectal	NA	16,380,321	NA	2021	Finngen [22]	M, F
finn-b-c3-bladder	Bladder	NA	16,380,305	NA	2021	Finngen [22]	M, F
finn-b-c3-larynx	Larynx	NA	16,380,304	NA	2021	Finngen [22]	M, F
finn-b-c3-lip-oral	Lip/Oral/Pharynx	NA	16,380,304	NA	2021	Finngen [22]	M, F
ieu-b-94	Oral cavity	4151	7,510,833	Lesseur	2016	OOCOC	M, F
finn-b-c3-brain	Brain	NA	16,380,308	NA	2021	Finngen [22]	M, F
finn-b-c3-Biliary tract	Biliary tract	NA	16,380,304	NA	2021	Finngen [22]	M, F
finn-b-c3-oesphagus	Oesphagus	NA	16,380,466	NA	2021	Finngen [22]	M, F

WFGC: Within Family GWAS Consortium. BACA: Breast Cancer Association Consortium; OOCOC: Oncoarray Oral Cavity and Oropharyngeal Cancer.

**Table 2:** Two-sample Mendelian randomization results between alcohol consumption and cancer site: comparison before and after outlier removal.

Cancer Site	Sample Size	Cases	Before Removing Outliers		After Removing Outliers	
			OR (95% CI)	*p* Value	OR (95% CI)	*p* Value
Stomach	174,006	633	1.06 (0.56–1.98)	0.86300	1.06 (0.73–1.54)	0.7739
Liver	218,488	304	1.14 (0.46–2.83)	0.7704	0.73 (0.27–1.94)	0.4401
Lung	217,165	2,671	0.99 (0.99–1.00)	0.4408	0.99 (0.99–1.00)	0.4584
Colon	174,006	1,803	1.13 (0.77–1.66)	0.5098	0.85 (0.62–1.16)	0.2978
Thyroid	174,006	989	1.18 (0.70–1.97)	0.5616	2.39 (2.07–2.74)	≤0.0001
Cervix	99,321	1,648	0.98 (0.62–1.55)	0.9260	0.98 (0.73–1.32)	0.9096
Breast	115,178	15,748	0.93 (0.81–1.63)	0.2660	0.90 (0.82–0.99)	0.0159
Prostate	74,865	6311	1.09 (0.80–1.48)	0.5630	1.05 (0.86–1.26)	0.6568
Colorectal	215,770	3022	0.94 (0.70–1.27)	0.6844	0.87 (0.72–1.06)	0.2467
Bladder	174,006	1115	1.29 (0.80–2.09)	0.2906	1.17 (0.99–1.38)	0.1062
Larynx	218,612	180	2.12 (0.44–10.13)	0.3357	NA	NA
Lip-Oral-Pharynx	218,666	126	1.16 (0.28–4.74)	0.8317	1.53 (0.64–3.62)	0.3661
Oral cavity	4151	1223	2.33 (0.91–5.92)	0.07685	NA	NA
Brain	218,328	464	0.98 (0.45–2.13)	0.9604	1.04 (0.65–1.67)	0.8965
Biliary tract	174,006	109	0.15 (0.01–3.97)	0.30220	0.60 (0.28–1.27)	0.3423
Oesophagus	218,560	232	1.61 (0.58–4.48)	0.3590	1.83 (0.90–3.75)	0.0449

Alcohol consumption (id: ieu-b-4834).

**Table 3:** Comparison of the two sample Mendelian randomization results between alcohol intake frequency and cancer sites.

Cancer Site	Sample Size	Cases	Before Removing Outliers		After Removing Outliers	
			OR (95% CI)	*p* Value	OR (95% CI)	*p* Value
Stomach	174,006	633	0.48 (0.23–1.00)	0.05	1.77 (0.97–3.23)	0.0036
Liver	218,488	304	0.96 (0.34–2.71)	0.9386	0.82 (0.33–2.07)	0.6813
Lung	217,165	1627	0.92 (0.58–1.45)	0.7169	1.08 (0.73–1.58)	0.7088
Colon	174,006	1803	1.11 (0.768–1.616)	5.69 × 10^−1^	1.83 (0.99–3.35)	0.0022
Thyroid	174,006	989	1.12 (0.57–2.18)	0.7411	1.15 (0.65–2.04)	0.6219
Cervix	99,321	1648	0.99 (0.54–1.80)	0.9665	0.85 (0.55–1.30)	0.4488
Breast	115,178	8401	0.84 (0.61–1.15)	0.2786	0.93 (0.84–1.02)	0.1189
Prostate	88,902	6311	0.99 (0.71–1.38)	0.9552	0.93 (0.72–1.21)	0.6027
Colorectal	215,770	3022	0.79 (0.57–1.11)	0.1822	0.85 (0.67–1.09)	0.1666
Bladder	174,006	1115	1.19 (0.69–2.05)	0.5414	1.08 (0.88–1.30)	0.5002
Larynx	218,612	180	0.59 (0.15–1.27)	0.4409	0.69 (0.19–2.45)	0.5743
Lip-Oral-Pharynx	218,666	126	1.14 (0.20–6.64)	0.8808	1.02 (0.28–3.78)	0.7621
Oral cavity	4151	1223	2.32 (0.91–5.92)	0.0768	NA	NA
Brain	218,328	464	1.35 (0.58–3.14)	0.4905	1.16 (0.57–2.36)	0.6839
Biliary tract	174,006	109	1.44 (0.25–8.43)	0.6776	3.86 (0.89–16.85)	0.0722
Oesophagus	218,560	232	2.58 (0.74–9.00)	0.1380	3.44 (1.19–9.89)	0.0217

## Data Availability

The data used in this study are publicly available and can be accessed by anyone. All datasets analyzed during this study are open at MR-Base platform (http://www.mrbase.org).

## Supplementary Materials

The following supporting information can be downloaded at: https://www.mdpi.com/article/10.3390/epidemiologia5030043/s1, Table S1: Two sample Mendelian Randomization between alcohol consumption and cancer sites (id:ieu-b-4834); Table S2: Two sample Mendelian Randomization between alcohol intake frequency and cancer sites (ukb-a-25).
